# Mixed-Ploidy and Dysploidy in *Hypericum perforatum*: A Karyomorphological and Genome Size Study

**DOI:** 10.3390/plants11223068

**Published:** 2022-11-12

**Authors:** Shaghayegh Mehravi, Ghasem Karimzadeh, Alaeddin Kordenaeej, Mehrdad Hanifei

**Affiliations:** 1 Department of Plant Genetics and Breeding, Faculty of Agriculture, Tarbiat Modares University, Tehran 14115-336, Iran; 2 School of Biological Sciences, University of Western Australia, Perth, WA 6009, Australia; 3 Department of Agronomy and Plant Breeding, Faculty of Agriculture, University of Shahed, Tehran 33191-18651, Iran

**Keywords:** *Hypericum perforatum*, karyotype variability, genome size, dysploidy, mixoploidy, polyploidy

## Abstract

Karyomorphology and genome size of 15 St John’s wort (*Hypericum perforatum* L.) populations are reported for the first time. Root tips and fresh young leaves were used for karyological studies and flow cytometric (FCM) measurements, respectively. The chromosome length varied from 0.81 µm to 1.16 µm, and chromosome types were determined as “m”. Eight different somatic chromosome numbers were found (2*n* = 16, 22, 24, 26, 28, 30, 32, 38). Based on the observed basic (*x*) chromosome numbers of *x* = 8, 11, 13, 14, 15, 19, this may correspond to diploid (2*x*), triploid (3*x*), tetraploid (4*x*), respectively. Interestingly, we found mixoploidy (3*x* − 4*x*) in the root tips of one of the populations. Hybridization, polyploidy and dysploid variation may be the main factors associated with the chromosome number evolution of this species. FCM showed that 2C DNA contents vary from 0.87 to 2.02 pg, showing more than a 2-fold variation. The mean amount of 2C DNA/chromosome and the mean of monoploid genome size were not proportional to ploidy.

## 1. Introduction

Chromosome number and structure changes play an important role in speciation events, adaptation, promoting macroevolution and the development of new genetic networks during evolution [1,2]. Chromosomal changes, including hybridization, polyploidization and dysploidy, may supply the cytological mechanisms for isolation, environmental adaptation and ecological differentiation between populations [3]. Accordingly, genome size and chromosome changes, their effects on species identification and speciation events and their ecological importance are the subjects of many studies in angiosperm [4,5].

Due to remarkable variation in the basic chromosome number and uneven geographical distribution, *Hypericum* is an outstanding genus to examine the role of chromosome variation in its diversification. *Hypericum* is the largest genus in the Hypericaceae family [6], includes approximately 490 species and has a cosmopolitan distribution [7]. Plants in this genus are annual or perennial herbs, subshrubs or shrubs and are distributed throughout tropical mountains, dry and warm sites in various types of grasslands, ruderal sites and disturbed habitats [8]. Within the genus *Hypericum*, *H. perforatum* L. is a medicinal plant that produces a wide range of secondary metabolites with antiviral activities [9]. *Hypericum perforatum* was originally distributed in Europe, Western Asia and North Africa, with a broad Eurasian native range extending from the Atlantic seaboard of Europe to China and India [10].

*Hypericum perforatum* is widespread across Europe, Asia, North America and consists of four subspecies, including *H. perforatum* subsp. *perforatum* (widespread and worldwide invasive) [11], *H. perforatum* subsp. *songaricum* (Ledeb. ex Rchb.) N. Robson (Asia) [12], *H. perforatum* subsp. *chinenese* N. Robson (China) [11] and *H. perforatum* subsp. *veronense* (Schrank) H. Lindb. (from Asia to the Mediterranean and Macaronesia) [12].

Since the chromosomes of *H. perforatum* are quite small, its karyotype structure is little available. Basic chromosome numbers that have been reported for the *Hypericum* species vary between the following: *x* = 6 for *H. perforatum* var. *angustifolium* DC. [13], *x* = 7 for *H. punctatum* Lam. [14], *x* = 8, 9, 12 and 16 for *H. tosaense* Makino [15]*, H. ascyron* L. [16], *H. perforatum* L. var. *angustifolium* DC. [13], *H. pseudopetiolatum* R. Keller [15] and *x* = 10 for *H. calycinum* L. [17]. Diploidy, triploidy, tetraploidy and hexaploidy were reported based on *x* = 6, 7, 8, 9, 10, 12 and 16 [10,11,15,18,19,20].

*Hypericum perforatum* is commonly a tetraploid species with the basic chromosome number of *x* = 8 [21], although diploid (2*x*), triploid (3*x*) and hexaploid (6*x*) plants occasionally have been identified [10,11,18]. According to the morphological traits of species and their geographic distribution, some authors [11,22] suggested that *H. perforatum* is derived by hybridization between the diploids *H. maculatum* subsp. *immaculatum* (Murb.) A.Fröhl. and *H. attenuatum* Fisch. ex Choisy and subsequent chromosome doubling [10]. On the other hand, karyotype analysis of *Hypericum* species revealed that this species may have arisen through auto-polyploidization from a diploid *H. maculatum* [23]. However, wild populations of *H. perforatum* are composed of diploids and polyploids, which were identified from glacial refuge areas in southern Europe, the Balkans and Japan [24]. It is noteworthy that different types of mixoploids (2*n* = 2*x* − 3*x*, 2*x* − 4*x*, 3*x* − 4*x*, 4*x* − 5*x*, 2*x* − 3*x* − 4*x*, 3*x* − 4*x* − 5*x* − 6*x*), were unambiguously observed, either karyologically or flows cytometrically [20,25,26,27,28]. Although some chromosome numbers were found recurrently, generally, it seems to be a swarm of possibly aneuploid forms (2*n* + 4 and 2*n* − 1), as reviewed by Brutovská et al. [25].

Due to shortcomings in some of the used cytogenetic techniques and according to the fact that the plant samples were partly unreliably recognized, some chromosome counts existing in the literature for *H. perforatum* should be treated with caution. So far, no detailed karyotype analysis is available for Iranian populations. In contrast, Iran is located in the center of the origins of diversity, so it is expected that Iranian *H. perforatum* germplasm indicates much of the worldwide genetic diversity of *H. perforatum.* Thus, reliable conclusions cannot be drawn on the actual range of chromosomal variation in *H. perforatum* without considering the Iranian germplasm.

Nuclear DNA content is a feature under strict genetic control and is widely applied in biological, biodiversity, systematic and evolutionary and plant breeding studies [2,29,30,31,32,33]. Intra- or inter-specific genome size differences are well known and expected, especially when it represents a karyotype change in chromosomal number and size [34]. Greilhuber et al. [29] described homoploid genome size (1 C-value) as the DNA content of the haploid chromosome complement (with chromosome number *n*) and monoploid genome size (1Cx-value) as the amount of DNA of one basic chromosome set (with chromosome base number *x*), regardless of the degree of generative polyploidy, aneuploidies, etc. Differences in chromosome number in the genus *Hypericum* can suggest intra- and inter-specific variation in nuclear DNA content. Brutovská et al. [25] reported 2C DNA = 1.55 pg for *H. perforatum* (2*n* = 4*x* = 32). The 2C DNA content of *Hypericum* species from various localities all over Europe and Japan demonstrated the range of genome size between 1.3 pg and 6.0 pg [26].

The present study aimed to provide a detailed survey of chromosomal and genome size variation in the Iranian *H. perforatum*. According to this aim (1) we made karyotype analysis and described chromosome numbers of 15 populations, focusing on populations that were not studied before, (2) we investigated numerical variation in monoploid chromosome numbers, checked for different ploidy levels and potential to illustrate the huge somatic chromosome number variations and (3) we estimated of genome size to find out whether there is a relationship between 2C DNA and monoploid genome size.

## 2. Results

### 2.1. Chromosome Counts, Basic Numbers and Ploidy Levels

**Figure 1:**
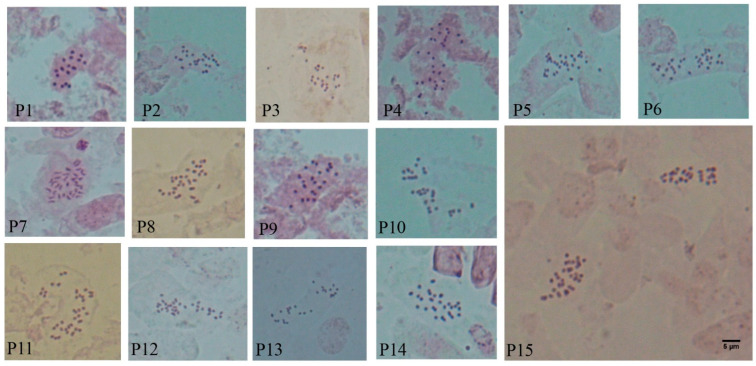
Somatic chromosomes of 15 *Hypericum perforatum* L. populations. Scale bar = 5 μm.

**Figure 2:**
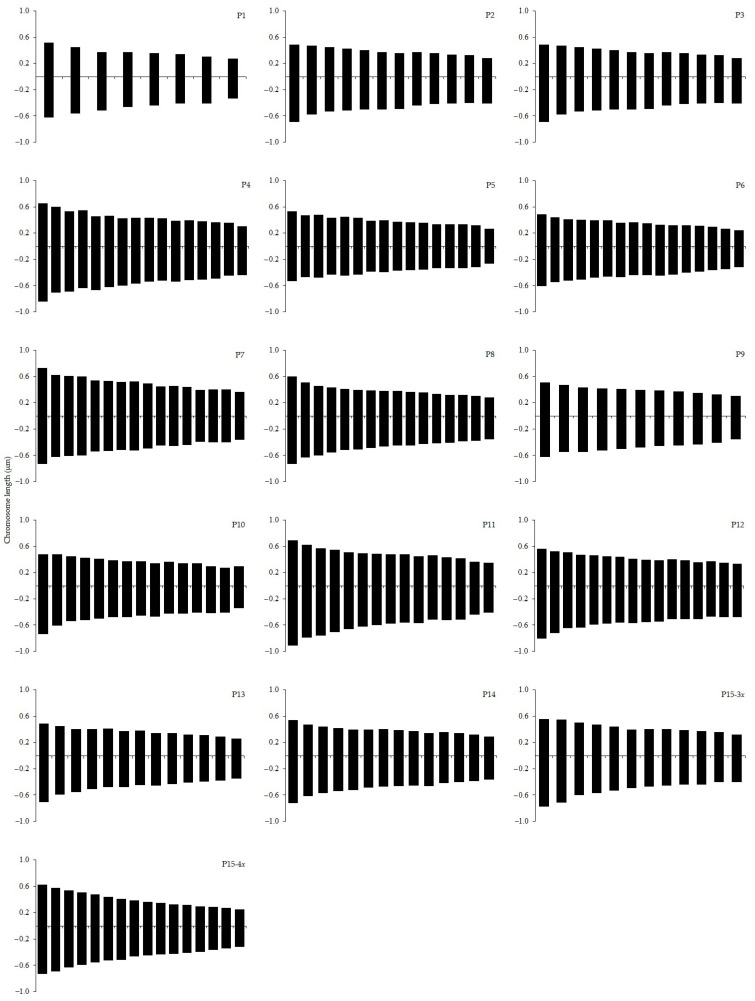
Haploid chromosome idiograms of 15 *Hypericum perforatum* populations.

Of the 15 *H. perforatum* populations, one (P1) had a chromosome number of 2*n* = 2*x* 16, 2 (P2 and P3) were determined as 2*n* = 3*x* = 24 and 5 (P4–P8) were determined as 2*n* = 4*x* = 32. For 6 populations, i.e., P9 (2*n* = 2*x* = 22), P10 and P11 (2*n* = 2*x* = 30), P12 (2*n* = 2*x* = 38), P13 (2*n* = 2*x* = 26) and P14 (2*n* = 2*x* = 28), chromosome numbers are reported for the first time. In 14 populations, all cells studied consistently had the same chromosome number. Variable chromosome numbers within the same individual were found (the most frequent being underlined in the following) in the P15 population (2*n* = 3*x* − 4*x* = 24, 32). Figure 1 and Figure 2 show the somatic complement karyotypes and haploid complement idiograms of the examined *H. perforatum* populations, respectively.

The most likely basic chromosome numbers (*x*) and ploidy levels were as follows: *x* = 8 in P1, which had 2*n* = 16, P2 and P3 with 2*n* = 3*x* = 24 and P4-P8 with 2*n* = 4*x* = 32 (Figure 1). *x* = 11 occurred in P9, *x* = 13 and 14 were found in P13 and P14, respectively. *x* = 15 was observed in P10 and P11 with 2*n* = 2*x* = 30. *x*= 19 occurred only in P12, which had 2*n* = 2*x* = 38. Therefore, the most frequent basic number of *x* = 8 was consistently found in 8 of the 15 populations of *H. perforatum*.

### 2.2. Karyotypes

The mean chromosome length was 0.88 and 0.89 µm in P15-3*x* and P15-4*x*, respectively. Using Levan et al. [35] chromosome nomenclature, one chromosome type, types “m” (centromere at median region), formed the following 8 different karyotype formulas: 2*n* = 2*x* = 16m (P1); 2*n* = 3*x* = 24m (P2, P3, P15-3*x*); 2*n* = 4*x* = 32m (P4, P5, P6, P7, P8, P15-4*x*), 2*n* = 4*x* − 2 = 30m (P10 and P11), 2*n* = 3*x* − 2 = 22m (P9), 2*n* = 5*x* − 2 = 38m (P12), 2*n* = 3*x* + 2 = 26m (P13) and 2*n* = 3*x* + 4 = 28m (P14) (Table 1).

Karyotype analysis with detailed measurements is presented in Table 1. The chromosomes of the genus *Hypericum* are small. The mean centromeric asymmetry (M_CA_) varied from 0.65 in P7 (4*x*) (more symmetrical chromosomes) to 0.78 in P9 (2*x*) with more asymmetrical chromosomes. Interchromosomal asymmetry (CV_CL_), indicating the variation of chromosome lengths within the whole complement, was lowest in P9 (CV_CL_ = 15.53) and highest in P1 (CV_CL_ = 26.63).

The karyotypes of 11 populations (P1, P2, P4-P6, P9, P10, P12-P15-3*x*) were classified as 1A, and five populations (P3, P7, P8, P11, P15-4*x*) were classified as 1B according to the Stebbins classification [36].

### 2.3. Regression Analysis

**Figure 3:**
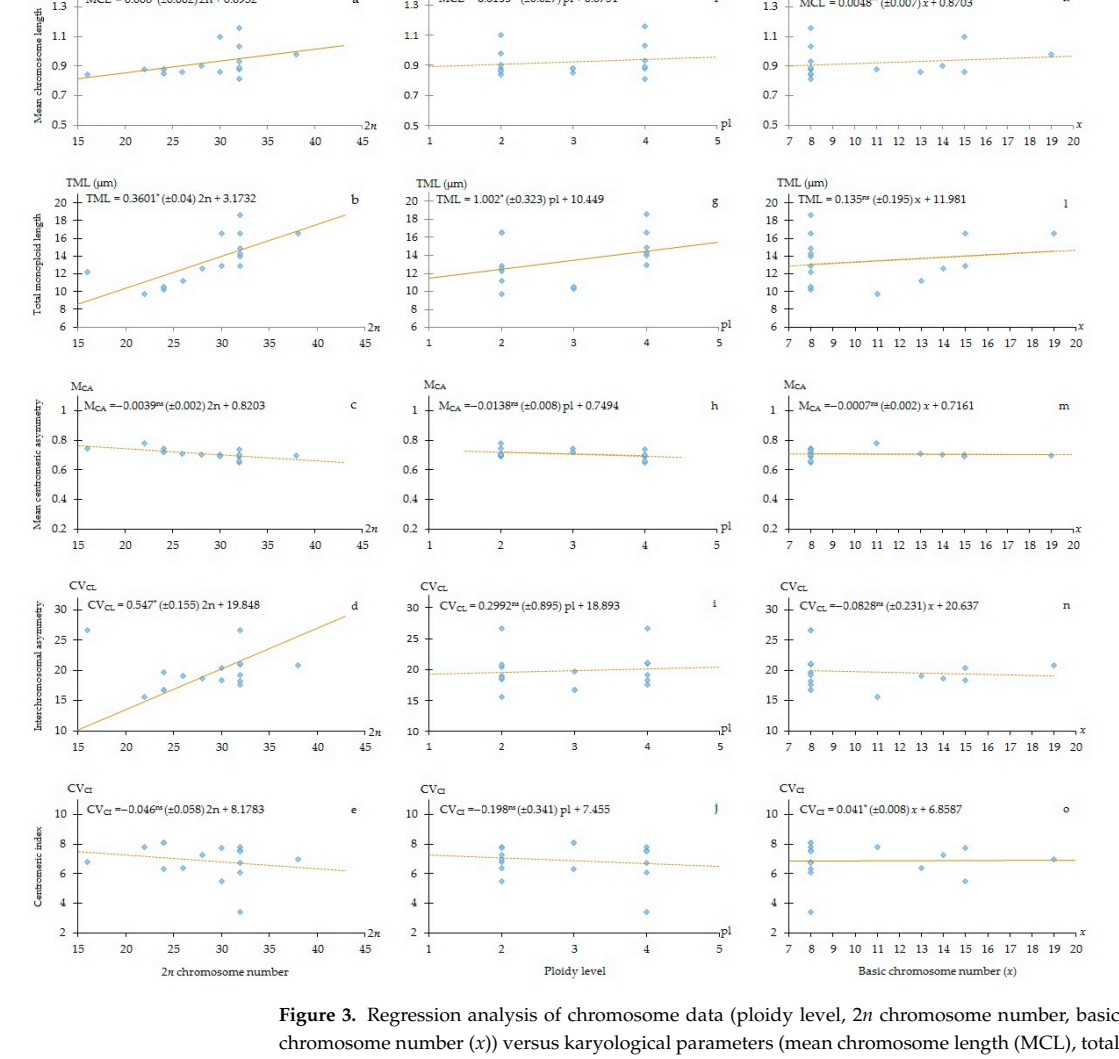
Regression analysis of chromosome data (ploidy level, 2*n* chromosome number, basic chromosome number (*x*)) versus karyological parameters (mean chromosome length (MCL), total length of monoploid chromosome set (TML); interchromosomal asymmetry (CV_CL_), centromeric index (CV_CI_) and mean centromeric asymmetry (M_CA_) in *Hypericum perforatum* populations. Solid lines in **a**,**b**,**f** show significant linear regression, and dotted lines in the other plots represent not significant correlations. The five significant regression models in **a**,**b**,**d**,**g**,**o** had *p* values < 0.05. **a**–**o**: linear regression relationships between dependent and independent variables.

In Figure 3, the results for the linear regression models are presented as scatter plots, which refer to the dependent (mean chromosome length, total monoploid length, mean centromeric asymmetry, centromeric index and interchromosomal asymmetry) and independent variables (2*n*, ploidy level and *x*). The following trends in karyotype evolution were retrieved: There is a significant positive linear relationship between 2*n* chromosome number and TML (Figure 3b), meaning that an increase in 2*n* is linked with an increase in TML. Furthermore, ploidy level was significantly related to TML (Figure 3g). However, there are no relationships between 2*n* and ploidy level with M_CA_, while a weak interrelation was observed between 2*n* and CV_CL_, which is caused mainly by polyploidization (Figure 3d). Therefore, chromosomes of a complement seem to converge in their length after whole genome duplication. Basic chromosome number (*x*) and MCL indicated a significant linear relationship (Figure 3k). Moreover, *x* revealed a weak relationship with CV_CI_ (3o), indicating that an increase in basic chromosome number is generally accompanied by a slight increase in centromere position heterogeneity (i.e., a decrease in centromere position homogeneity) in karyotypes. There were no significant relationships between *x* and TML and CV_CL_. In addition, there is a weak relationship between *x* and M_CA_ (3m), indicating a general increase in intrachromosomal karyotype asymmetry (i.e., a general shift towards more terminal positions, concerning centromeres).

### 2.4. Principal Coordinate and Cluster Analysis

**Figure 4:**
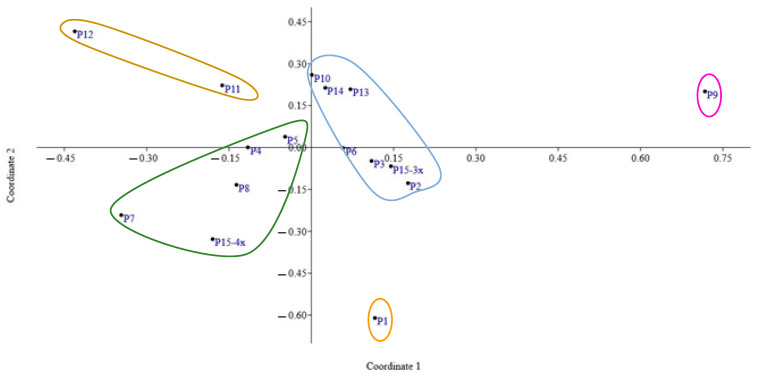
The scatter diagram of 15 *Hypericum perforatum* populations based on the first two coordinate of principal coordinate (PCoA) of the karyological parameters.

To determine the total variation in populations and parameter quotas, principal coordinate analysis (PCoA) was performed, showing that the first two PCo account for 66.01% of the cumulative variation. The first two coordinates were projected in a 2-dimensional graphic (Figure 4). The results showed that the parameters of basic chromosome number (−0.61), haploid chromosome length (−0.38) and chromosome number (0.11) showed stronger associations with the first coordinate, which accounted for 55.12% of the variations in the computed data. M_CA_ (0.33), haploid chromosome length (0.27) and CV_CI_ (0.19) played the most important quotas in the second coordinate, explaining 10.89% of the total variation. In summary, on the basis of karyological parameters, tetraploid populations (P4-P8, except for P6) with the same chromosome number (2*n* = 4*x* = 32) clustered together. The P6 population was separated based on the lowest haploid chromosome length. P10 and P11 populations had similar chromosome numbers (2*n* = 2*x* = 30), but were different in CV_Cl_ and CV_CI_ and therefore classified into different groups. Moreover, relating to chromosome number and karyological parameters, P1 and P9 are separated from other populations and stand in a single cluster. Hence, this clustering correctly separate populations and refers to chromosome number, ploidy level and karyological parameters.

### 2.5. Flow Cytometry

**Figure 5:**
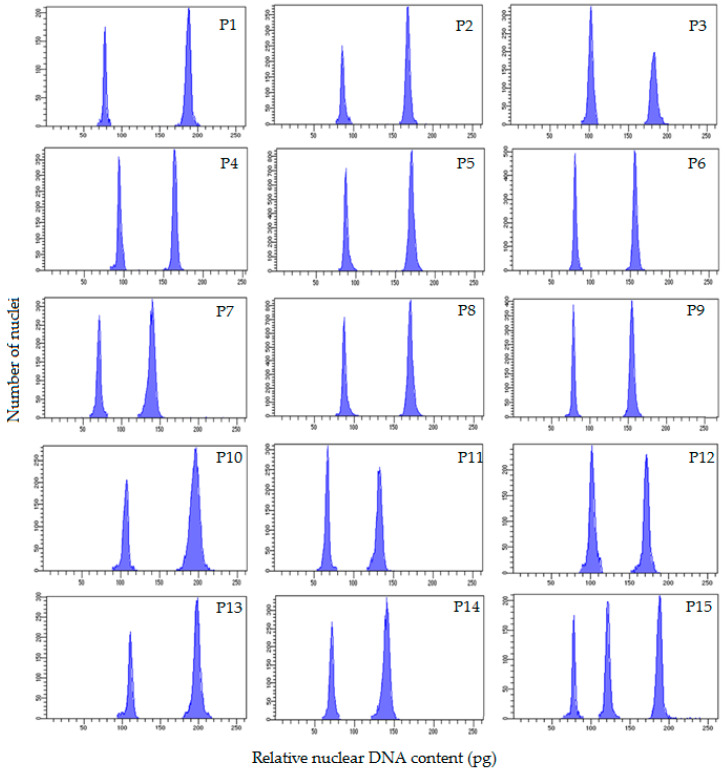
Histograms of nuclear 2C DNA content of 2*x* (**P1**, **P9**–**P14**), 3*x* (**P2**, **P3**), 4*x* (**P4**–**P8**) and 3*x* − 4*x* (mixoploid, **P15**) *Hypericum perforatum* populations. The left peaks refer to the G1 peaks of the sample and the right peaks refer to the G1 peaks of *Glycine max* (L.) Merr. cv. Polanka (2C DNA= 2.50 pg) as an internal reference standard plant (**P1**–**P14**). In **P15**, the right peak refers to the G1 peak of the reference standard plant.

The nuclear DNA values of fifteen populations of *H. perforatum* were estimated by flow cytometry. There were two peaks in the histograms obtained for the evaluation of the nuclear DNA amount in the leaves (except for P15, Figure 5).

**Figure 6:**
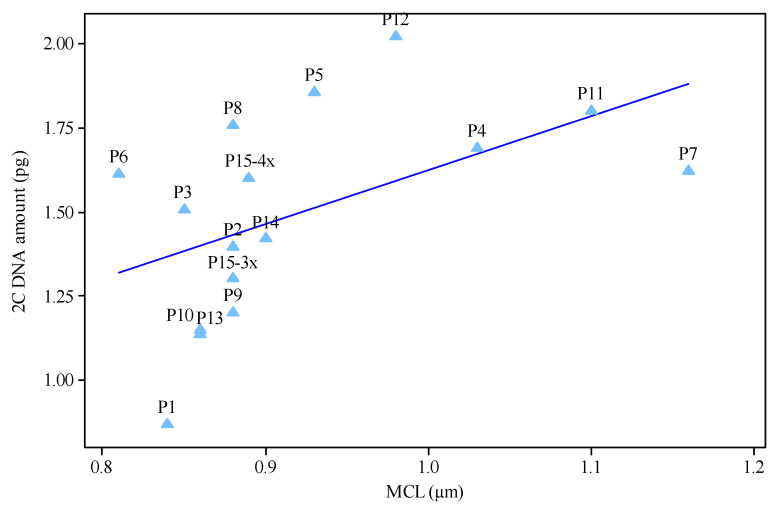
Linear relationship between 2C DNA amount (pg) and chromosome length (b = 1.25 ** *p* ˂ 0.01) of 15 *Hypericum perforatum* populations.

**Figure 7:**
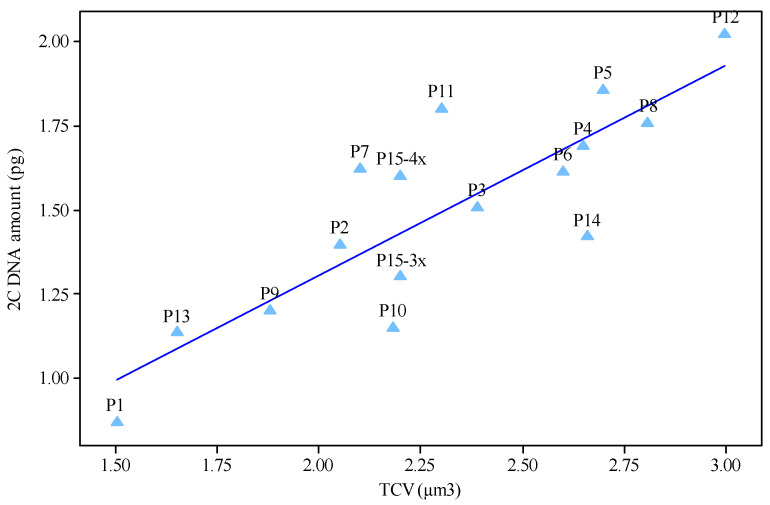
Linear relationship between 2C DNA amount (pg) and chromosome volume (b = 0.72 ** *p* ˂ 0.01) of 15 *Hypericum perforatum* populations.

In P1-P14 populations, the left peak corresponds to the *H. perforatum* populations and the right peak corresponds to the *Glycine max* (L.) Merr. cv. Polanka (2C = 2.50 pg) internal reference standard. In P15, three peaks were observed in the obtained histograms for measuring genomic DNA value. The left peak belongs to the triploid (3*x*) complements, the middle peak belongs to the tetraploid (4*x*) complements, and the right peak belongs to the *Glycine max* reference standard. The ANOVA indicated significant differences in nuclear 2C DNA amount among diploids, triploids and tetraploids populations, verifying intraspecific genome size variation (data not shown). The coefficients of variation of G1 peaks in either *H. perforatum* samples or internal reference standards varied between 1.4 and 2.1%. The 2C-values ranged from 0.87–2.02 pg (referring to diploids P1 and P12, respectively). Among seven diploids with a difference of 22 chromosomes (16 vs. 38), a difference of 1.15 pg in the 2C-value was recognized (Table 2). The mean 2C-value in the triploid and tetraploid populations were estimated as 1.45 and 1.71 pg, respectively. Among the two triploids examined, a small difference of 0.11 pg in the 2C-value (which varied from 1.40 in P2 to 1.51 in P3) was recognized. Despite having identical chromosome numbers, the five tetraploid populations showed differences up to 0.25 pg in their 2C-value (range 1.61–1.86). P6 and P5 were found to have the lowest and largest amounts of nuclear DNA within the tetraploids, respectively. On the other hand, the average 2C-value content of a P5 tetraploid (2*n* = 4*x* = 32, 1.86 pg) was 2-fold more than that of the P1 diploids (2*n* = 2*x* = 16, 0.87 pg). Among the chromosomal parameters studied, MCL and TCV revealed significant regression models with a 2C-value (Figure 6 and Figure 7).

## 3. Discussion

### 3.1. Variation of Chromosome Numbers in H. perforatum

Chromosome numbers in 15 different populations of *H. perforatum* reveal somatic chromosome consistencies in 5 populations. In 10 populations, we found chromosome numbers deviating from the tetraploid number. In our study, a consistent somatic chromosome number occurred in 5 out of the 15 examined populations. Variation in chromosome number was found in nine populations. In eight of them, two to eight different chromosome numbers were observed (Table 1). We consider the most frequently occurring somatic chromosome number of the species to establish the monoploid chromosome number and ploidy level (2*x*, 3*x*, 4*x*).

Root tips of plants with different somatic chromosome numbers often have uniform euploid chromosome numbers at meiosis and mostly form bivalents during the meiotic prophase [37,38]. Because of the scarcity of flowering in glasshouse cultures of *H. perforatum*, reliable information on the meiosis of the genus *Hypericum* is scarce in the literature. According to our findings, we assume that the chromosome numbers are variable, at least within the generative tissues.

Variation in somatic chromosome numbers has been reported in the root tips of many plants [2,3,39,40,41,42]. It was suggested that it is either an outcome of genome rearrangements such as fusions or fissions after recent polyploidization [39,43,44] or an outcome of irregular segregation of chromosomes as a consequence of meiotic abnormalities caused by somatic irregularities, namely, the presence of precocious chromosome migration to the poles or laggard chromosomes and of non-oriented bivalents at the equatorial plate [45,46].

### 3.2. Dysploidy Caused the Considerable Chromosome Number Variation

Our study demonstrates the occurrence of considerable somatic chromosome number variation in *H. perforatum*. The assumed basic chromosome numbers and the deduced ploidy levels of the examined populations are summarized in Table 1. Surveys of chromosomal indicated eight somatic chromosome numbers, namely, 2*n* = 16, 22, 24, 26, 28, 30, 32 and 38, which are considered as multiples of *x* = 8, 11, 13, 14, 15 and 19 as the likely basic chromosome numbers of the examined populations. Therefore, populations with these specific somatic chromosome numbers may correspond to diploids, triploids and tetraploids, correspondingly.

Matzk et al. [26] provided an explanation for the wide variation of chromosome numbers in the genus *Hyperiicum* by the investigation of the mode of reproduction and genome size. They presented a network of all possible chromosome numbers. Nevertheless, it should be considered that for the production of capable hybrids, crossing must be performed between populations with similar karyotypes [47]. However, hybridization is rather frequent in the genus *Hypericum*, as shown by intraspecific and interspecific studies [10]. Moreover, due to the facts that species of genus *Hypericum* (i) propagate mainly by apomixes, (ii) seed formation after flowering is very low [48] and (iii) the origin of chromosomal biotypes through sexual reproduction is fairly impossible [49], we assume that hybridization play only an ancillary role in the chromosome evolution of examined populations in our study. There were two cytogenetically verified cases of triploidy, in which meiotic disturbances cannot be excluded. In other populations, it is more likely that dysploidy, through gains and losses of chromosomes or fission and/or fusion of chromosome segments, is the most common mechanism of karyotypic variation in the genus *Hypericum*.

Polyploidy, through the beginning of reproductive isolation due to differential visitation and pollination between diploids and the established polyploids, is considered the most common chromosomal mechanism involved in plant speciation [50,51,52,53]. Accordingly, it played a significant role in the diversification and evolution of *H. perforatum*.

The wide variation of assumed basic chromosome numbers found in this study supports the evolutionary importance of karyotype changes via dysploidy, which may have comparatively longer-term perseverance over evolutionary time than polyploid changes that fail in many cases to persist [40]. Our findings in *H. perforatum* populations are in contrast to the attention polyploidy usually found in the literature. While the evolutionary role of polyploidy has been stressed in many reviews, chromosomal change through dysploidy has not been considered [40,54,55]. Up to now, the role of dysploidy in species diversification and its evolutionary effect have been demonstrated in *Chrysosplenium* [56], *Houstonia* [57], *Phaseolus* [58], *Cyananthus* [59], Microlepidieae [60], *Phalaris* [61], *Helianthemum squamatum* L. [62], Marantaceae family [3] and *Pulmonaria* [63,64]

In the current study, a mixoploid population composed of triploid and tetraploid was recorded for the first time in *H. perforatum*. It is assumed that mixoploidy is capable of significantly increasing the adaptive potential of plants, especially under unfavorable growth conditions and increasing the probability that cells with a changed number of chromosomes will occur, thereby creating conditions for the emergence of hybrids and polyploids and altering the reproductive pathways from zygotic to parthenogenetic [65]. The phenomenon of mixoploidy has also been described in the meristem tissues of the Cruciferae family in nature [66]. Knowledge of the prevalence of mixoploidy in the natural plant populations helps us arrive at a convenient estimate of the possibility and the cause of spontaneous hybridization between different species.

### 3.3. Chromosome Structure

Our data on chromosome morphology are important to understand the variation in chromosome numbers and to identify potentially different genomes within the genus *Hypericum*. Only one study reported karyotype characteristics of the genus *Hypericum* [67]. The results of karyotype analysis indicate that the chromosomes are overall comparatively small (<1.2 µm in length). Variations of chromosome length (CV_CL_) and mean centromeric asymmetry (M_CA_) are variable within the studied populations. In general, although the karyotypes of the studied populations are relatively continuous, they reveal significant variation.

Regression analyses of basic chromosome number (*x*), ploidy level (pl) and chromosome number (2*n*) versus karyotype data, such as total length of a monoploid chromosome set (TML), mean chromosome length (MCL), interchromosomal asymmetry (CV_CL_), centromeric index (CV_CI_), and mean centromeric asymmetry (M_CA_), respectively, were conducted (Figure 3). The negative relationship between *x* and CV_CI_ may be explained by hypothesizing an ancestral karyotype with a predominance of telocentric chromosomes. Under this picture, a fusion of two telocentric chromosome couples may cause a decrease in *x* but also an increase in the heterogeneity of centromere position among chromosomes. This is further supported by the weak positive correlation between CV_CI_ and M_CA_ (data not shown). However, the fact that the correlation among these two parameters is 0.41 further highlights how what CV_CI_ is measuring is different from intrachromosomal asymmetry, as already demonstrated by Zuo and Yuan [68]. A non-significant relationship between TML and CV_CL_ suggests that an increase in karyotype dimensions is not accompanied by changes in size between the chromosomes of the karyotype. Since no relationship was also found between M_CA_ and TML, this implies that the additional DNA is added mainly following a pattern of ‘proportional increase’ [69], i.e., the amount of DNA added to each chromosome arm is proportional to its length, not causing a change in karyotype asymmetry. In Liliaceae, Peruzzi et al. [70] found a low correlation between CV_CI_ and CV_CL_, highlighting the relevant role of Robertsonian translocations in the karyotype evolution of this family. The presence of such correlation (also between M_CA_ and CV_CL_) in *H. perforatum* confirms also in this species a relevant role for Robertsonian translocations, where indeed different dysploid basic chromosome numbers occur.

In this study, the direction of dysploidy change cannot be determined in the studied populations. However, there are the following two presumptions: either decreasing dysploidy accompanied by increasing chromosome length or, alternatively, increasing dysploidy accompanied by decreasing chromosome length. The first presumption causes fewer and larger chromosomes and is a frequent presumption in monocots following polyploidization [3]. To identify the original basic chromosome number in studied populations, a comparison of chromosome data with a molecular phylogenetic framework would be essential.

We applied PCoA to different populations of *H. perforatum*, showing that the discriminatory power of karyological parameters is very strong among populations. As already highlighted by Peruzzi and Altınordu [71], PCoA is best suited to establish karyological relationships, relationships, compared with classification approaches, which may be misinterpreted concerning their real significance. Moreover, the resolution of karyological relationships is much better than that obtained by simply plotting karyotype asymmetry parameters against each other [72]. Karyological data significantly contributes to understanding evolutionary relationships. However, basic karyological data alone are not sufficient to definitely establish systematic and phylogenetic relationships among taxa and should always be complemented by independent sources of systematic data [73,74,75].

### 3.4. Genome Size Differentiation

Flow cytometric analyses have been used successfully to identify the stability ploidy level in different plants [31,76,77,78,79,80,81,82,83,84]. The results of the present study showed that the amount of nuclear DNA provides a good tool to segregate different *H. perforatum* populations, show intra-species variations and verify the karyological results. The present data on the 2C DNA amount is in agreement with the previous investigation in *Hypericum*, which estimate the 2C DNA values of 2*n* = 2*x* = 0.78 pg (*H. perforatum*), 2*n* = 2*x* = 2.8 pg (*H. ascyron* L.), 2*n* = 3*x* = 2.6 pg (*H. balearicum* L.), 2*n* = 4*x* = 1.55 pg (*H. perforatum*), 2*n* = 4*x* = 2.1 pg (*H. elegans* Stephan ex Willd.), 2*n* = 5*x* = 1.8 pg (*H. xylosteifolium* (Spach) N. Robson) and 2*n* = 4*x* + 4 = 2.5 pg [25,26]. A surprising finding in 2C DNA value estimation is that the two *H. perforatum* populations with different chromosome numbers of 24 and 26, and two different estimations of the 2C value were collected from the same locality (Ziarat) with similar habitat conditions. The following major question remains unanswered: why are the two different *H. perforatum* populations from the same province different by one chromosome pair? Are there any genetic and ecological attributes that lead to different chromosome numbers? Systematic investigation into different aspects of this problem needs to be taken up. The 2C DNA value mean and monoploid genome size mean comparison among different ploidy levels were not statistically significant. In other words, such an analysis revealed that the 2C DNA averages and monoploid genome size averages were not matchable to ploidy in the examined *H. perforatum* populations, a relation, which is in accordance with the prevailing downward direction in genome size evolution in the angiosperms [85]. By studying six accessions of Iranian *Thymus* with different ploidy levels, Mahdavi and Karimzadeh [77] described smaller genome sizes in tetraploids compared with diploids. In some instances, the differences were not great, emphasizing that this reflected the nature of allopolyploidization in *H. perforatum*. Ozkan et al. [86] postulated that a reduction in genome size was associated with the adaptation necessary for the establishment and stabilization of allopolyploidy in *Triticum aestivum* L.

A significant relationship between MCL and 2C-value (b = 1.25 ** *p* ˂ 0.01) may indicate that karyotypes between populations in this species are relatively homogeneous. Relationship between chromosomal parameters and 2C DNA value has been reported in *Vicia* [87], *Thymus* species [77] and *Tulipa* [88]. There was also a positive linear relationship between 2C-value and TCV (b = 0.72 ** *p* ˂ 0.01). A significant positive relationship between genome size and TCV has been shown repeatedly between populations of the same species [77,88]. As nuclear DNA content increases, total chromosome volume increases and consequently cell sizes increase, which may induce seed sizes to become larger [80]. Populations that produce small seeds in greater numbers, may lead to greater dispersal ability. The large-genome populations, and thus large-seeded ones, may develop a set of characteristics that improve such negative consequences. In any case, the ability to disperse into new environmental conditions may participate to the reduced risk of extinction of small-genome populations and improve the probability of speciation occurrences [30].

## 4. Materials and Methods

### 4.1. Plant Materials

**Figure 8:**
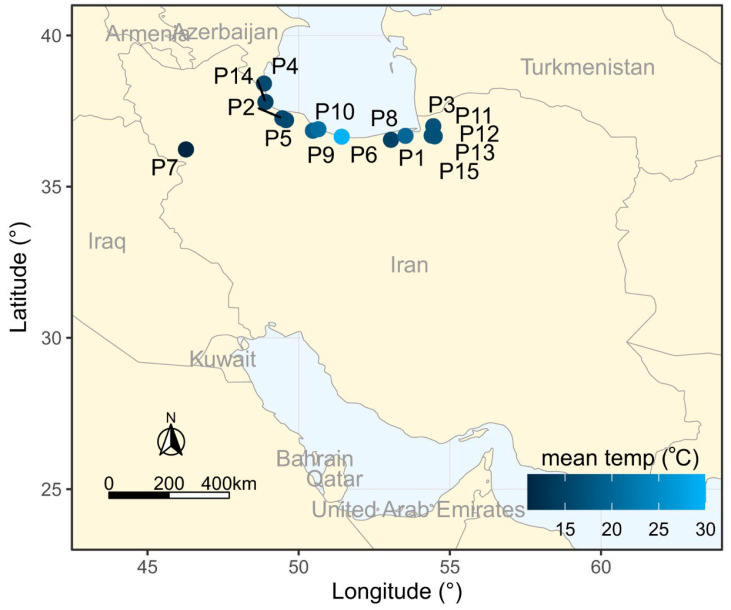
Location of the sampling sites of 15 Iranian endemic *Hypericum perforatum* L. populations on the map of Iran, using ArcGIS.

Root tips were excised from *Hypericum perforatum* plants of the living collection originated from different locations in Iran as described in Table 3 and Figure 8.

### 4.2. Karyologic Analysis

For the study of somatic chromosomes, 1–1.2 cm long fresh root tips were pretreated in 0.002 M 8-hydroxyquinoline solution for 5 h in darkness at room temperature. Roots were fixed in Carnoy’s fixative (glacial acetic acid: ethanol; 3:1 *v*/*v*) for 60 min at 4 °C [79,80]. After fixing, Carnoy’s fixative was removed by washing with distilled water, and meristems were hydrolyzed with 1M HCl at 60 °C for 7 min and then stained in 1% (*v*/*v*) Aceto-orcein at room temperature for 2 h [80]. Finally, the stained meristems were squashed in a driblet of 45% (*v*/*v*) acetic acid. For chromosomal measurements, five well-spread metaphase plates were used from different individuals per population. Photographs of metaphase plates from each individual were taken using a BX50 Olympus microscope equipped with a DP12 digital camera (Olympus). Different chromosomal parameters were determined, including short arm (S) long arm (L), chromosomes length (CL = L + S), total chromosome volume (TCV), arm ratio (AR), r-value, centromere index (CI), form percentage of chromosome (F%) and relative length of chromosome (RL%). Their formula was calculated as follows:

CL = L + S(1)

TCV = πr^2^ × CL (2)

(3) $$\mathrm{AR}=\frac{L}{S}$$

(4) $$r\text{-}\mathrm{value}=\frac{S}{L}$$

(5) $$\mathrm{CI}=\frac{S}{\mathrm{CL}}$$

(6) $$\mathrm{RL}\%=\frac{\mathrm{CL}}{\sum \mathrm{CL}}\times 100$$

(7) $$F\%=\frac{S}{\sum \mathrm{CL}}\times 100$$

where L and S are long and short arms lengths and CL, π and “r” are chromosome length, the ratio of a circle’s circumference (=3.14), and the average chromosome radius, respectively.

Idiograms were constructed using chromosome types were determined, based on the formula of Levan et al. [35]. For karyotype analysis, three distinct methods including coefficient of variation of chromosome length (CV_CL_, formula 8), mean centromeric asymmetry (M_CA_, formula 9) and coefficient of variation of centromeric index (CV_CI_, formula 10) were used to evaluate the degree of karyotype asymmetry. Their formulae are as follows Paszko [89] and Peruzzi and Eroğlu [74]:

(8) $${\mathrm{CV}}_{\mathrm{CL}}\%=\frac{S_{\mathrm{CL}}}{X_{\mathrm{CL}}}\times 100$$

(9) $$M_{\mathrm{CA}}=\frac{\sum_{i=1}^{n}\frac{L-S}{L+S}}{n}$$

(10) $${\mathrm{CV}}_{\mathrm{CI}}\%=\frac{S_{S/L+S}}{X_{S/L+S}}\times 100$$

### 4.3. Flow Cytometric (FCM) Analyses

Around one cm^2^ of young and well-developed leaf from each population together with a half area of leaf material from *Glycine max* (L.) Merr. cv. Polanka (2C DNA = 2.50 pg) as internal standard were chopped (1 min) into small pieces in 1 mL of woody plant buffer, supplemented with 50 µL of Propidium Iodide (PI, Fluka) as DNA staining agent and 50 µL of RNase solution (Sigma-Aldrich Corporation, MO, USA). Then, the resultant nuclear suspension was passed through a 30 µm Partec green nylon mesh and analyzed using a BD FACSCanto II flow cytometer (BD Biosciences, Bedford, MA, USA), equipped with a 532 nm green high-grade solid-state laser. Analyses were carried out on five (*n* = 5) individuals per population using a BD FACSDiva^TM^ Software. Histograms with a coefficient of variation (CV) in the range between 3.20 and 4.50% for G1 peaks were evaluated. The 2C DNA (pg) content was estimated using the linear relationship between the ratio of the G1 peak means of the target population/G_1_ peak mean of the internal standard × Standard 2C DNA (pg).

### 4.4. Statistical Analyses

The cytological and flow cytometric data were tested for normality and then were analyzed based on completely randomized design (CRD) with five replicate cells. Homoploid nuclear DNA content (1C-value) was estimated based on a conversion factor suggested by Doležel et al. [90], where 1 pg = 978 Mbp. Multivariate statistical analysis was carried out in the Past software (Past ver. 4.03) [91]. To investigate karyological relationship among populations, a principal coordinate analysis (PCoA) analysis on chromosome number, basic chromosome number, total haploid chromosome length, CV_CL_, M_CA_ and CV_CI_ was performed [71]. To perform PCoA analysis, a similarity matrix was created using Gower’s [92] general coefficient similarity to summarize relationships among populations which can be used directly with a mixture of character types (binary, qualitative, and quantitative characters) as well as taking into account missing values [93]. For regression analysis, linear models were calculated between the five dependent variables MCL, TML, M_CA_, CV_CI_ and CV_CL_ and the three predictor variables ploidy level (pl), chromosome number (2*n*) and basic chromosome number (*x*) using the software Excel 2013. The regression equation is presented on each plot with the following parameters: an estimate of the regression slope, its standard error, the *t* test statistic and the two-sided significance level for the null hypothesis of regression slopes equal to zero. Moreover, the linear regression analysis was performed to examine the relationship between the mean of 2C-value as predictor variables and TCV, MCL as dependent variables.

## 5. Conclusions

Our results have added important information on the number and morphological properties of the chromosomes of the *Hypericum perforatum* populations. This contributes to a better understanding of the chromosome changes that have occurred during the karyotype diversification of this species. Our study has also confirmed that total chromosome volume is positively correlated to genome size, and both increases and decreases in genome size may have had a role in the evolution and diversification of the genus, even within a closely related group of species. Our finding may provide remarkable information for *H. perforatum* evolution.

## Figures and Tables

**Table 1:** Somatic chromosome number, ploidy level, basic chromosome number and mean chromosomal and karyological parameters of 15 *Hypericum perforatum* populations. S: small arm length; L: long arm length; CL: total length of chromosome; AR: arm ratio; RL: relative length of chromosome; F%: form percentage of chromosome; TCV: total chromosome volume; CI%: centromeric index; M_CA_: mean centromeric asymmetry; CV_CL_: interchromosomal asymmetry; CV_CI_: coefficient of variation of centromeric index; KF: Karyotype formula.

Population	pl	2*n*	*x*	S (µm)	L (µm)	CL (µm)	AR	r-Value	RL%	F%	TCV (µm^3^)	CI%	M_CA_	CV_CL_	CV_CI_	KF
P1	2*x*	16	8	0.37	0.47	0.84	1.27	0.80	12.50	5.53	0.25	44.22	0.74	26.63	6.79	16m
P2	3*x*	24	8	0.39	0.49	0.88	1.29	0.79	8.33	3.67	0.31	44.03	0.74	16.73	8.08	24m
P3	3*x*	24	8	0.37	0.48	0.85	1.29	0.78	8.33	3.64	0.30	43.75	0.72	19.68	6.31	24m
P4	4*x*	32	8	0.45	0.58	1.03	1.31	0.77	7.81	3.39	0.53	43.39	0.69	19.18	7.80	32m
P5	4*x*	32	8	0.39	0.54	0.93	1.39	0.73	7.83	3.30	0.27	42.11	0.69	18.21	7.56	32m
P6	4*x*	32	8	0.35	0.45	0.81	1.28	0.79	7.78	3.43	0.34	44.05	0.74	17.58	6.74	32m
P7	4*x*	32	8	0.50	0.66	1.16	1.30	0.78	7.77	3.38	0.53	43.67	0.65	20.91	7.49	32m
P8	4*x*	32	8	0.39	0.49	0.88	1.25	0.81	7.79	3.48	0.39	44.65	0.66	21.17	6.05	32m
P9	2*x*	22	11	0.40	0.48	0.88	1.22	0.83	9.09	4.11	0.55	45.19	0.78	15.54	7.79	22m
P10	2*x*	30	15	0.38	0.48	0.86	1.30	0.79	7.92	3.47	0.38	43.85	0.70	18.36	7.74	30m
P11	2*x*	30	15	0.49	0.61	1.10	1.25	0.81	7.89	3.51	0.45	44.64	0.69	20.40	5.51	30m
P12	2*x*	38	19	0.43	0.55	0.98	1.32	0.77	7.58	3.29	0.39	43.40	0.70	20.88	6.96	38m
P13	2*x*	26	13	0.38	0.48	0.86	1.26	0.80	8.14	3.61	0.35	44.39	0.71	19.04	6.40	26m
P14	2*x*	28	14	0.40	0.50	0.90	1.26	0.81	8.02	3.56	0.34	44.51	0.70	18.71	7.27	28m
P15	3*x*	24	8	0.39	0.49	0.88	1.29	0.79	8.33	3.67	0.30	44.03	0.72	16.73	8.08	24m
	4*x*	32	8	0.40	0.49	0.89	1.23	0.81	7.60	3.42	0.40	44.83	0.70	26.61	3.39	32m

**Table 2:** 2C DNA value (pg) of 15 *Hypericum perforatum* populations.

Population	Ploidy Level	2*n*	2C-Value (pg ± SE)	1C-Value (pg)	1C*x*-Value (pg)
P1	2*x*	16	0.87 ± 0.021	0.435	0.435
P2	3*x*	24	1.40 ± 0.018	0.700	0.470
P3	3*x*	24	1.51 ± 0.018	0.755	0.503
P4	4*x*	32	1.69 ± 0.021	0.845	0.423
P5	4*x*	32	1.86 ± 0.020	0.930	0.465
P6	4*x*	32	1.61 ± 0.022	0.805	0.403
P7	4*x*	32	1.62 ± 0.022	0.810	0.405
P8	4*x*	32	1.76 ± 0.022	0.880	0.440
P9	2*x*	22	1.20 ± 0.020	0.600	0.600
P10	2*x*	30	1.15 ± 0.021	0.575	0.575
P11	2*x*	30	1.80 ± 0.032	0.900	0.900
P12	2*x*	38	2.02 ± 0.022	1.010	1.010
P13	2*x*	26	1.13 ± 0.025	0.565	0.565
P14	2*x*	28	1.42 ± 0.021	0.710	0.710
P15	3*x*	24	1.30 ± 0.030	0.650	0.433
	4*x*	32	1.60 ± 0.030	0.800	0.400

**Table 3:** Geographical description of Iranian endemic *Hypericum perforatum* populations.

Population Codes	Local Collection Sites	Latitude (N)	Longitude (E)	Altitude (m)	Mean Temp. (°C)	Mean Rainfall (mm)
P1	Sari, Mazandaran, Iran	36°33′	53°03′	43	15	2122
P2	Rasht, Gilan, Iran	37°16′	49°34′	−3	15.9	1359
P3	Aq Qala, Golestan, Iran	37°00′	54°27′	−14	17	675
P4	Astara, Gilan, Iran	38°25′	48°51′	−26	16	1500
P5	Lakan, Gilan, Iran	37°12′	49°35′	19.5	15.9	1359
P6	Chalus, Mazandaran, Iran	36°39′	51°25′	24	30	700
P7	Baneh, Kordestan, Iran	36°14′	46°16′	1496	11	386
P8	Behshahr, Mazandaran, Iran	36°41′	53°32′	23	20	977
P9	Javaherdeh, Mazandaran, Iran	36°51′	50°28′	1772	19	1200
P10	Ramsar, Mazandaran, Iran	36°54′	50°39′	36	21	1200
P11	Ziarat, Golestan, Iran	36°42′	54°28′	918	17	649
P12	Ziarat, Golestan, Iran	36°42′	54°28′	918	17	649
P13	Aq Qala, Golestan, Iran	37°00′	54°27′	−14	17	675
P14	Talesh, Gilan, Iran	37°48′	48°54′	42	15	1446
P15	Ziarat, Golestan, Iran	36°42′	54°28′	918	17	649

## Data Availability

The data presented in this study are available on request from the corresponding author.
